# Age as a biological stratifier for immune checkpoint inhibitor response in ovarian cancer

**DOI:** 10.21203/rs.3.rs-10655967/v1

**Published:** 2026-09-14

**Authors:** Tirzah Braz Petta, Kathy Kwock, Lynda Roman, Joseph W. Carlson

**Affiliations:** University of Southern California; University of Southern California; University of Southern California; City of Hope National Medical Center

**Keywords:** ovarian cancer, immune checkpoint inhibitor, aging, STING1, IFI16, cGAS, BRCA, immunotherapy biomarker, innate immunity

## Abstract

**Background.:**

Immune checkpoint inhibitor (ICI) monotherapy produces response rates of only 8–15% in ovarian cancer, widely considered ICI-refractory. However, DNA damage sensing and interferon signaling undergo pronounced age-dependent remodeling, making patient age a plausible modifier of ICI response.

**Methods.:**

We integrated four ovarian cancer transcriptomic cohorts (n = 761) and tested the age-by-BRCA–immune interaction by pre-specified random-effects meta-analysis, comparing Young (< 50 y) versus Old (≥ 60 y) patients with the peri- and early post-menopausal transition (50–59 y) excluded from primary comparisons. Age-dependent expression of cGAS, STING1, IFI16, and IDO1 was quantified. A single-institution ICI-treated cohort (USC/LAGMC, n = 20; Responder defined as progression-free interval ≥ 365 days) provided clinical anchoring.

**Results.:**

Patient age reversed the BRCA–immune relationship across all four cohorts (pooled interaction b = −0.275, 95% CI - 0.502 to - 0.048, p = 0.018, I^2^=0%). STING1 was reduced in older patients across three platforms (TCGA-OV FDR = 0.014; GSE9891 p = 0.0026; GSE140082 p = 6.5×10^−7^), together with the nuclear DNA sensor IFI16 (TCGA-OV FDR = 0.018; GSE140082 p = 0.007), while upstream cGAS was unchanged (p = 0.771). Downstream IDO1 was concurrently reduced (p = 0.009). Tumor mutational burden, HRD status, BRCA1 methylation, and an eight-gene checkpoint panel did not account for the age signal. Clinically, patients ≥ 60 y achieved 75% response versus 14% in patients < 50 y (Fisher p = 0.041), independent of BRCA status.

**Conclusions.:**

Patient age is a biological determinant of the ovarian cancer immune microenvironment through selective STING1/IFI16 attenuation with cGAS preserved. These findings support a systematic reanalysis of existing ovarian cancer ICI clinical trial data stratified by patient age.

## Introduction

Ovarian cancer remains the most lethal gynecologic malignancy in the United States, with an estimated 20,890 new diagnoses and 12,730 deaths in 2025 alone^1^. Median age at diagnosis is 63 years, and mortality is concentrated in the 65–74 age group. Immune checkpoint inhibitor (ICI) therapy — which has transformed outcomes across other cancers — has produced only 8–15% single-agent objective response rates in ovarian cancer clinical trials^2-4^. This modest performance has led to the widely held view that ovarian cancer is intrinsically refractory to ICI, with subsequent trial designs emphasizing combination regimens or de-emphasizing this indication altogether.

A critical enrollment feature has been overlooked. The median patient age in ICI clinical trials in ovarian cancer is approximately 60 years^2-3^ — within or just before the peri- and early post-menopausal transition range (50–59 years), and substantially below both the U.S. median age at diagnosis (63 years) and the 65–74 year peak in mortality. Post-menopausal women, who carry most of the disease burden, have been systematically underrepresented in trials from which ICI shows low efficacy. This is not a minor sampling issue: enrollment falls squarely within a biological transition zone during which immune cell composition, T cell dynamics, and regulatory T cell function undergo nonlinear remodeling driven by hormonal decline^12^. Trials concentrated in this transition window mix biologically distinct pre- and post-menopausal immune states in unknown proportions, obscuring effects present in one state but not the other.

We addressed this by pre-specifying a study design that isolates biologically distinct pre- and post-menopausal states rather than averaging across the transition. All analyses dichotomize patients into a Young (< 50 years) pre-menopausal group and an Old (≥ 60 years) post-menopausal group, with the intervening 50–59 year transition range excluded from primary comparisons^12^. The exclusion is methodological, not selective: 50–59 year patients are processed identically and contribute to descriptive cohort characterization. If the disease has been trialed predominantly in the transition range plus the younger tail while the true post-menopausal responder subpopulation was underrepresented, the prevailing view that ICI is ineffective in ovarian cancer would be a trial-design artifact rather than a biological property of the disease. We test this possibility directly.

Two considerations make patient age a mechanistically plausible modifier of ICI response. Ovarian cancer is defined by pervasive genomic instability and HRD, both of which generate cytosolic double-stranded DNA and single-stranded DNA gaps at stalled replication forks. These lesions are sensed by cGAS and IFI16, transduced through STING1 (TMEM173), and drive type I interferon signaling that shapes anti-tumor T cell recruitment^5-7^ — age-dependent attenuation of this axis would decouple DNA damage from immune recognition. Separately, the BRCA pathway sits at the center of clinical decision-making in ovarian cancer through PARP inhibitor selection, yet whether BRCA pathway activity relates uniformly to immune infiltration across patient age has never been tested.

We hypothesized that patient age is an active biological determinant of the ovarian cancer immune microenvironment through selective attenuation of innate immune signal transduction. To test this, we integrated four independent public ovarian cancer transcriptomic cohorts spanning RNA-seq, Affymetrix microarray, and Illumina DASL FFPE platforms (n = 761, four institutions)^8-11^ with an ICI-treated clinical cohort (USC/LAGMC, n = 20) with per-patient age, TNM stage, BRCA status (germline or somatic), HR status, and progression-free-interval-based response classification. Here we report that patient age qualitatively reverses the BRCA–immune relationship, replicated across all four cohorts with zero heterogeneity in formal meta-analysis. The molecular basis is a discrete lesion: STING1 and IFI16 are reduced in older patients across independent platforms while cGAS remains unchanged, and downstream IDO1 is concurrently reduced. Clinically, patients ≥ 60 years achieved 75% ICI response versus 14% in patients < 50 years (Fisher p = 0.041), independent of BRCA status and HR status. These findings reframe the prevailing view of ICI ineffectiveness as a consequence of age-unstratified trial design.

## Results

### Patient age determines ICI response in the USC/LAGMC ovarian cancer cohort

**Figure 1:**
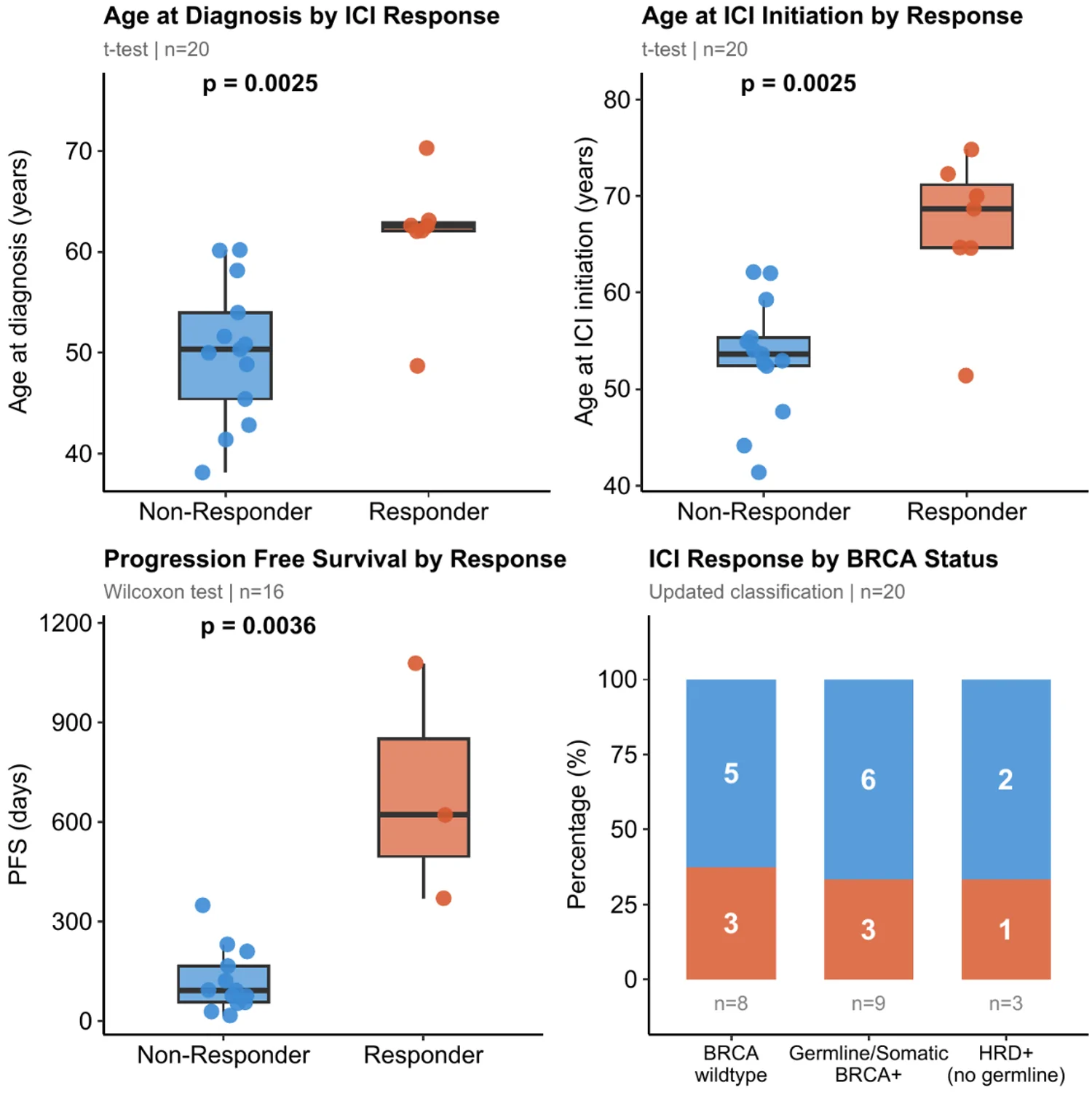
Patient age determines ICI response in the USC/LAGMC ovarian cancer cohort (n=20). (a) Cohort overview showing age at diagnosis and BRCA classification for all 20 patients. (b) Response rate by age group (Responder defined as PFI ≥ 365 days): patients ≥60 years (n=8) versus <50 years (n=7); Fisher exact p=0.041. (c) Progression-free survival by age group (Kaplan–Meier). (d) Response by BRCA subgroup: wildtype (n=8), germline/somatic BRCA-mutant (n=9), and HRD-positive without germline mutation (n=3); 38% of BRCA wildtype patients respond. Blue: non-responder; orange: responder.

We first assessed whether patient age was associated with ICI response in a fully clinically annotated single-institution cohort. Twenty women with histologically confirmed ovarian cancer treated with ICI at USC Norris Comprehensive Cancer Center and Los Angeles General Medical Center (USC/LAGMC) were included; all specimens were formalin-fixed paraffin-embedded (FFPE), with individual age at diagnosis, TNM stage, germline or somatic BRCA1/BRCA2 mutation status, HR status, and progression-free-interval-based response classification (Responder defined as PFI ≥ 365 days) available for every patient (Fig. 1a).

Response rates were markedly different between age groups. Patients ≥ 60 years at diagnosis (n = 8) achieved a 75% objective response rate (6/8 responders), while patients < 50 years (n = 7) responded at only 14% (1/7; Fisher exact p = 0.041; Fig. 1b). Progression-free survival was correspondingly longer in the older group (Fig. 1c). The age effect was not confounded by germline BRCA status: response occurred across all genomic subgroups, and notably 38% (3/8) of germline BRCA wildtype patients responded, a proportion incompatible with a model in which BRCA loss and homologous recombination deficiency are prerequisite for ICI benefit (Fig. 1d). HR status did not independently predict response in this cohort (see Section on competing hypotheses below).

### The BRCA–immune axis reverses with patient age across four independent transcriptomic cohorts

**Figure 2:**
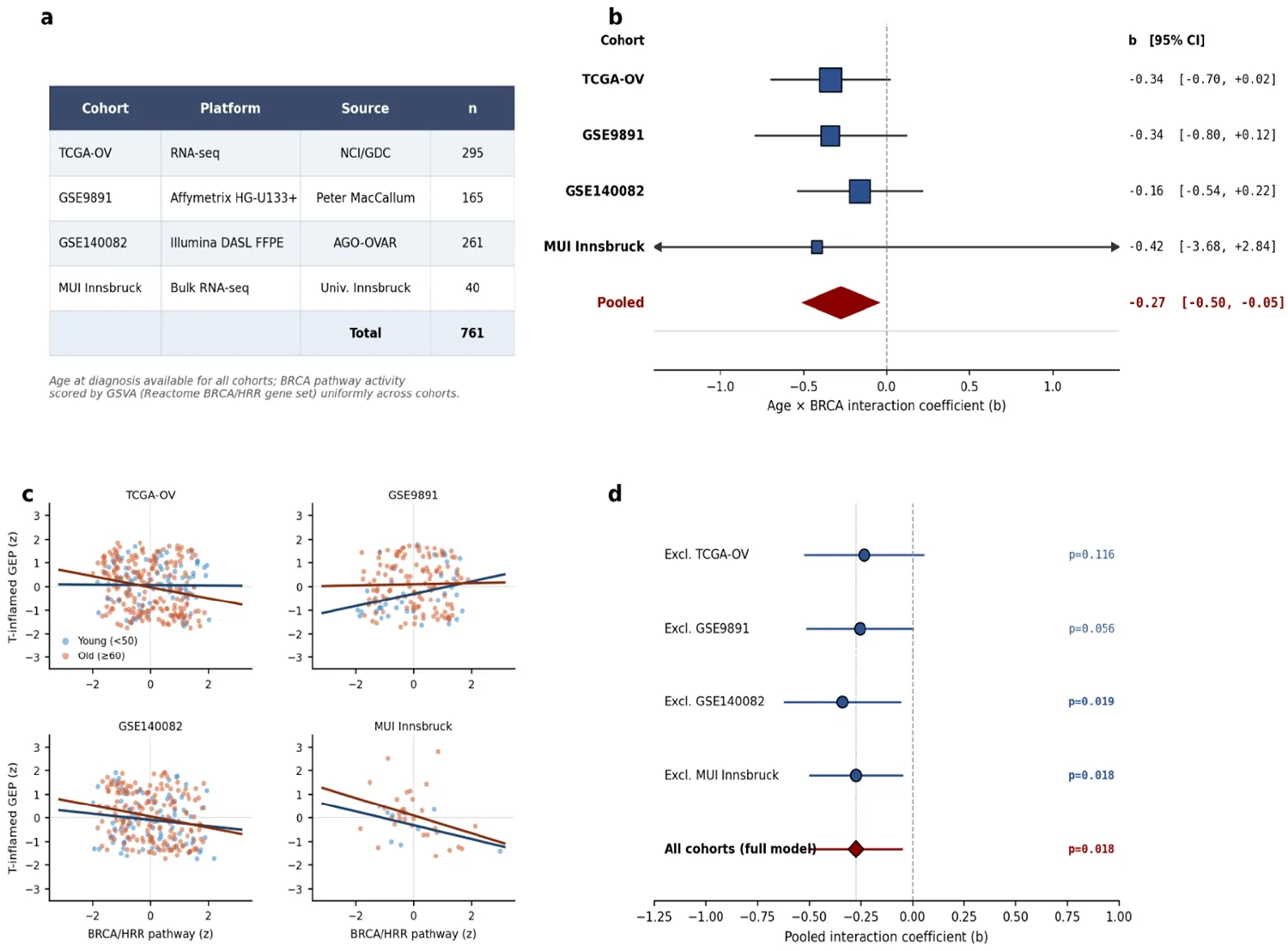
Meta-analysis reveals age-dependent reversal of the BRCA–immune axis in ovarian cancer (n=761). (a) Cohort table listing the four independent transcriptomic cohorts, platforms, sample sizes, and institutional origins. (b) Forest plot of the age-by-BRCA interaction coefficient across cohorts; pooled random-effects estimate b=−0.275 (95% CI: −0.502 to −0.048), p=0.018, I^2^=0%. (c) Per-cohort scatterplots of BRCA pathway activity versus immune infiltration score, stratified by age group; the direction of the correlation reverses between Young and Old patients across all cohorts. (d) Leave-one-out sensitivity analysis showing that no single cohort accounts for the pooled result.

The clinical observation in a single-institution cohort of n = 20, while striking, cannot on its own establish either the biological mechanism underlying the age-dependent response or its generalizability beyond one institution. To determine whether the age effect reflects a reproducible molecular signal at the population level, and to identify a candidate mechanism that could be tested prospectively in expanded clinical cohorts, we turned to four independent public transcriptomic cohorts (n = 761 across three platforms and four institutions; Fig. 2a, Table 1): TCGA-OV RNA-seq^8^; the Australian Ovarian Cancer Study Peter MacCallum cohort profiled on Affymetrix HG-U133 Plus 2.0 (GSE9891)^9^; the AGO-OVAR cohort profiled on Illumina DASL FFPE arrays (GSE140082)^10^; and the Medical University of Innsbruck (MUI) RNA-seq cohort^11^. All cohorts contained age at diagnosis and expression measurements needed to score BRCA pathway activity by GSVA and immune infiltration.

For each cohort, we tested a linear model of immune infiltration score against BRCA pathway activity, patient age (dichotomized as Old ≥ 60 years vs. Young < 50 years, with the peri- and early post-menopausal transition range (50–59 years) excluded to isolate biologically distinct pre- and post-menopausal immune states^12^), and their interaction. Pooling the four cohorts by random-effects meta-analysis yielded a highly significant age-by-BRCA interaction coefficient of b = - 0.275 (95% CI: −0.502 to −0.048, p = 0.018), with zero heterogeneity across cohorts (I^2^=0%; Fig. 2b). Per-cohort scatterplots confirmed that the direction of the BRCA–immune correlation was reversed between age groups, with older patients displaying a decoupling or inversion of the BRCA-high/immune-high relationship characteristic of younger patients (Fig. 2c). Leave-one-out sensitivity analyses confirmed that no single cohort was responsible for the pooled result (Fig. 2d), and the interaction remained significant after adjustment for tumor purity, stage, and platform.

The convergence of RNA-seq, microarray, and DASL FFPE platforms on the same age-by-BRCA interaction, with I^2^=0%, indicates that the phenomenon is a robust biological signal rather than a technical artifact of any single measurement modality.

### STING1 and IFI16 are attenuated in older ovarian cancer patients while cGAS is preserved

To identify the molecular basis of the age-by-BRCA interaction, we examined age-dependent expression of the canonical DNA-damage-sensing pathway in ovarian cancer transcriptomic cohorts. STING1 (TMEM173) is the central signal transducer of cytosolic double-stranded DNA sensing initiated upstream by cGAS, and additionally receives input from the nuclear DNA sensor IFI16, which detects single-stranded DNA gaps at stalled replication forks. Attenuation of any node in this pathway would decouple DNA damage — which is pervasive in ovarian cancer— from downstream type I interferon signaling and T cell recruitment.

**Figure 3:**
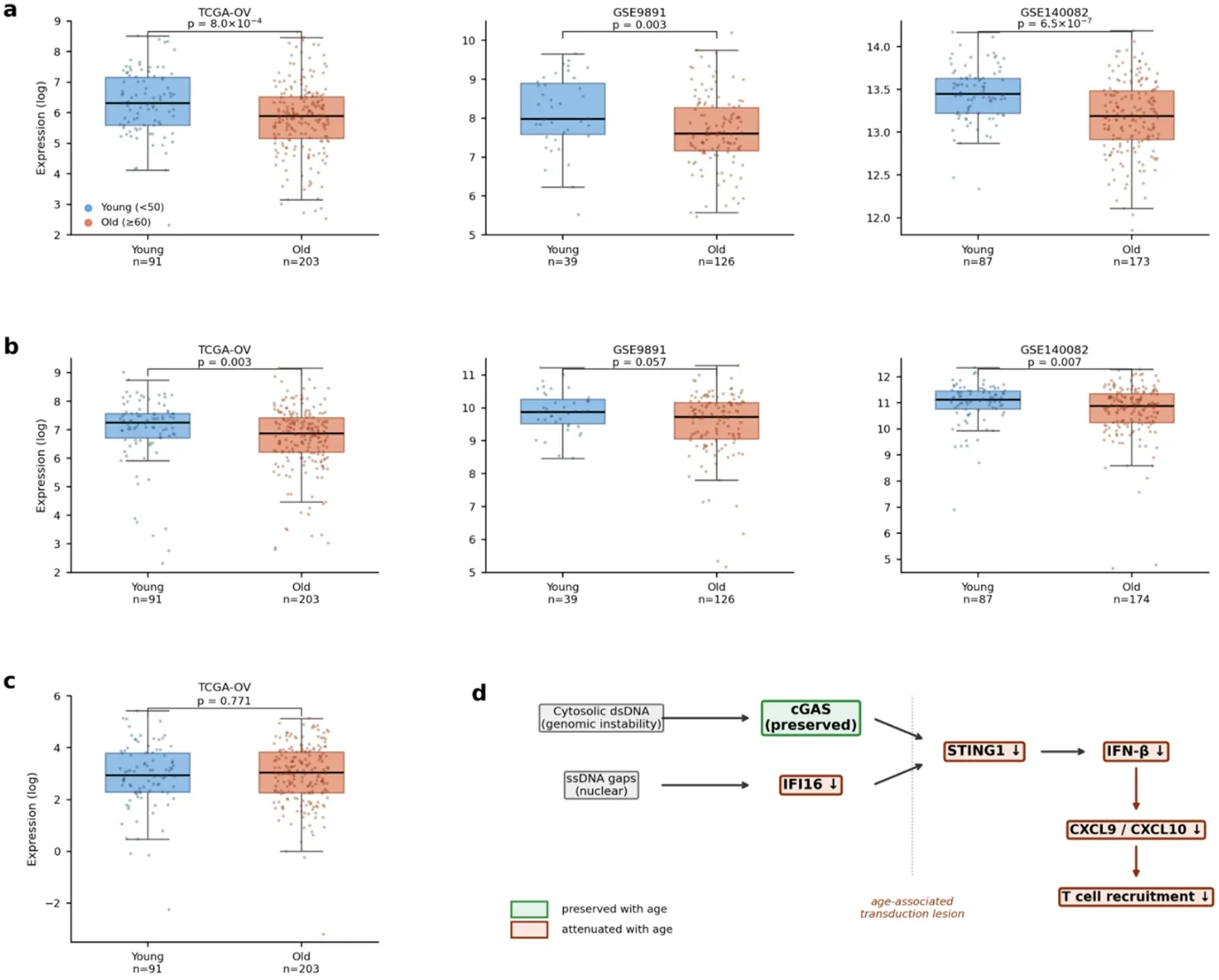
STING1 and IFI16 are attenuated in older ovarian cancer patients while cGAS is preserved. (a) STING1 (TMEM173) expression by age group across three independent ovarian cancer cohorts: TCGA-OV (RNA-seq, FDR=0.014); GSE9891 (Affymetrix, p=0.0026); GSE140082 (Illumina DASL FFPE, p=6.5×10^−7^). Age groups: Young <50 yr vs. Old ≥60 yr; 50–59 yr excluded. Wilcoxon rank-sum test. (b) IFI16 expression by age group across the same three cohorts (TCGA-OV FDR=0.018; GSE140082 p=0.007; GSE9891 p=0.057). (c) cGAS expression by age group in TCGA-OV (p=0.771; unchanged). (d) Schematic of the DNA damage sensing pathway showing the compartment of the age-associated lesion: cGAS sensing preserved, STING1/IFI16 transduction attenuated.

STING1 was significantly reduced in older patients across three independent ovarian cancer platforms (TCGA-OV: FDR = 0.014; GSE9891: p = 0.0026; GSE140082: p = 6.5×10^−7^; Spearman ρ ≈ −0.26 consistently across cohorts; Fig. 3a). The direction, magnitude, and statistical significance were highly concordant across platforms with distinct measurement biases (RNA-seq, microarray, DASL FFPE), supporting a genuine biological effect.

IFI16 was similarly reduced with age (TCGA-OV FDR = 0.018; GSE140082 p = 0.007; GSE9891 p = 0.057, trend; Fig. 3b), with consistent directionality across cohorts and an effect size approximately half that observed for STING1. The convergent reduction of two independent innate DNA sensors argues against a single-gene stochastic effect and instead points to coordinated age-associated remodeling of the innate immune signal transduction machinery.

In striking contrast, cGAS — the upstream cytosolic DNA sensor — was unchanged with age (TCGA-OV p = 0.771; Fig. 3c). The dissociation between preserved sensing (cGAS intact) and attenuated transduction (STING1 and IFI16 reduced) localizes the age-associated lesion downstream of DNA damage recognition and upstream of interferon output. This selectivity distinguishes the phenomenon from generic immunosenescence, in which broad decline of immune function is expected, and instead points to a discrete molecular lesion that would be expected to blunt interferon-driven T cell attraction while leaving the sensing of DNA damage intact (Fig. 3d).

Prior work has established that tumor-cell–intrinsic STING is recurrently silenced across cancers as an immune-evasion mechanism, frequently through promoter methylation, with pan-cancer analyses across 32 tumor types showing that the cGAS–STING axis is commonly inactivated in the tumor compartment specifically^13^. Our observation extends this framework by identifying patient age as a systematic modifier of STING1 expression in ovarian cancer — a stratifier that has not been considered in prior mechanistic studies of the pathway.

### Decoupling of the DDR–cGAS–STING–IDO1 circuit in older ovarian cancer patients

STING1-driven type I interferon output regulates a network of downstream effectors, including the immunometabolic enzyme indoleamine 2,3-dioxygenase 1 (IDO1). IDO1 catalyzes tryptophan degradation in the tumor microenvironment and is a well-characterized mediator of T cell metabolic suppression, with IDO1-high tumors typically displaying an exhausted rather than excluded T cell phenotype. We examined whether the age-dependent attenuation of STING1 and IFI16 propagates to IDO1 and whether the DDR–cGAS–STING–IDO1 circuit remains coupled with age.

**Figure 4:**
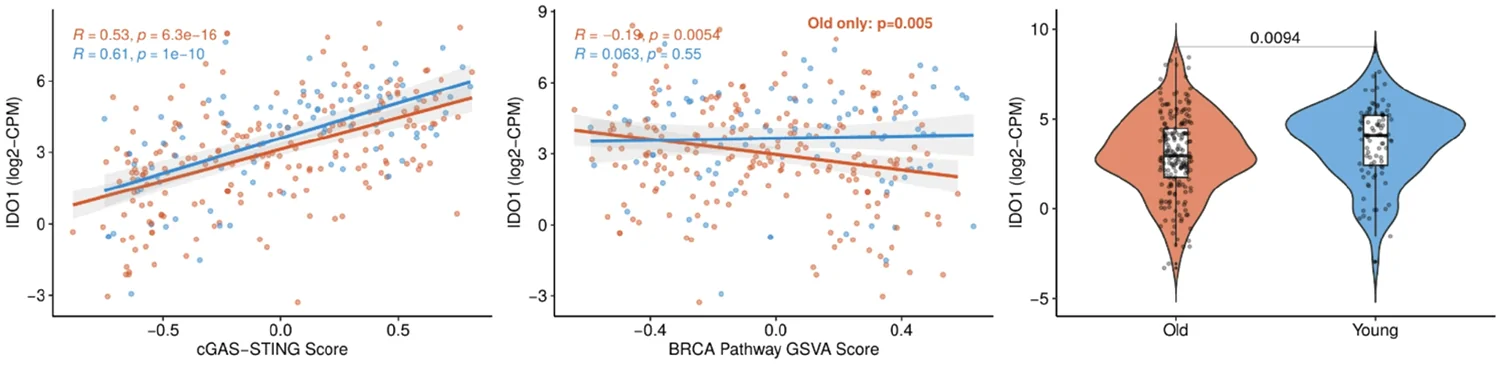
Decoupling of the DDR–cGAS–STING–IDO1 circuit with age. (a) IDO1 expression correlates with the cGAS–STING signaling score in both age groups (Old: R=0.53, p=6.3×10^−16^; Young: R=0.61, p=1×10^−10^). (b) Age-specific inverse relationship between IDO1 and BRCA pathway GSVA score in older patients only (Old: R=−0.19, p=0.005; Young: R=0.063, p=0.55; interaction p=0.005). (c) IDO1 expression by age group; IDO1 is significantly higher in younger patients (p=0.009).

In TCGA-OV, IDO1 expression correlated strongly with the cGAS–STING signaling score in both age groups (Old: R = 0.53, p = 6.3×10^−15^; Young: R = 0.61, p = 1×10^−10^; Fig. 4a), consistent with intact IDO1 regulation by cGAS–STING at the correlation level. Critically, however, the age-specific relationship of IDO1 to the BRCA pathway inverted: in older patients, IDO1 was inversely correlated with BRCA pathway GSVA score (Old: R = - 0.19, p = 0.005), while in younger patients this relationship was absent (Young: R = 0.063, p = 0.55; interaction p = 0.005; Fig. 4b). Overall, IDO1 expression was significantly lower in older than in younger patients (p = 0.009; Fig. 4c).

This pattern implies a compartment- and age-specific reconfiguration of the DDR–cGAS–STING–IDO1 circuit. In younger patients, high IDO1 in the tumor microenvironment is expected to drive T cell metabolic exhaustion through tryptophan depletion, consistent with the exhausted phenotype typically encountered in metabolically hostile tumors. In older patients, the concurrent reduction in STING1, IFI16, and IDO1 produces a distinct configuration: reduced tryptophan-depleting activity (low IDO1) combined with attenuated type I interferon-driven CXCL9/CXCL10 recruitment^14^. This molecular reconfiguration is consistent with the age-dependent ICI response phenotype observed clinically.

### Systematic exclusion of competing predictors of ICI response

**Figure 5:**
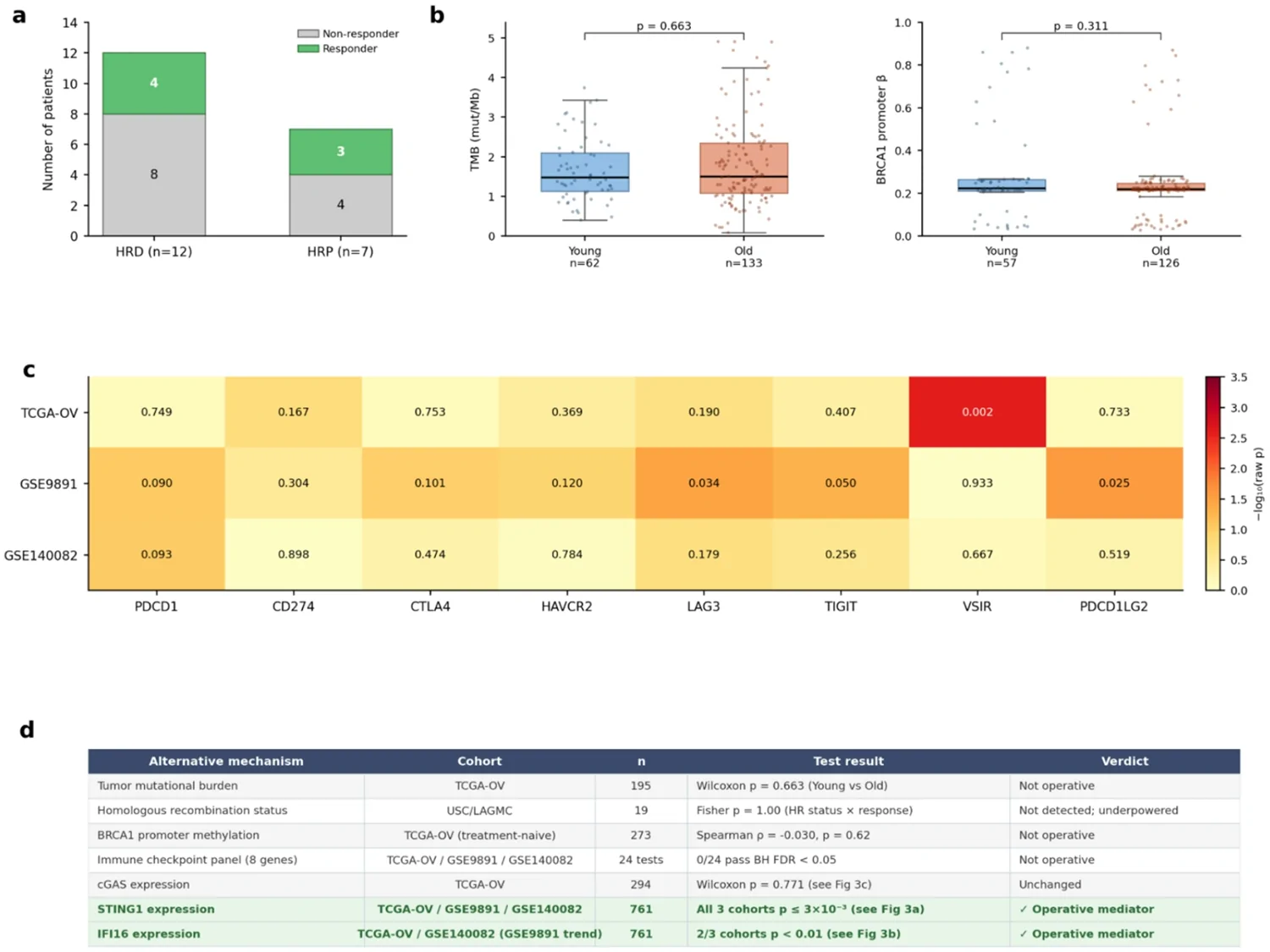
Systematic exclusion of competing predictors of ICI response and the age-dependent immune configuration. (a) HR status does not predict ICI response in the USC/LAGMC cohort (n=19 with HR annotation; Fisher exact p=1.0; underpowered at n=20). (b) In TCGA-OV, tumor mutational burden does not differ by age group (Wilcoxon p=0.663, n=195), and BRCA1 promoter methylation β-values in the treatment-naive subset (n=273 patients from 282 arrays, all GDC-verified as primary tumor) do not correlate with age (Spearman ρ=−0.030, p=0.62). (c) An eight-gene immune checkpoint expression panel (PDCD1, CD274, CTLA4, HAVCR2, LAG3, TIGIT, VSIR, PDCD1LG2) across three ovarian cancer cohorts (24 Wilcoxon tests; TCGA-OV, GSE9891, GSE140082) shows no differential expression surviving BH FDR<0.05 (0/24). VSIR raw p=0.0025 in TCGA-OV (BH FDR=0.059) did not replicate in the microarray cohorts. (d) Summary table of predictors tested and their verdicts; only STING1 with IFI16 as secondary contributor satisfies the pre-specified criteria for a primary mediator.

To determine whether STING1 and IFI16 attenuation is the primary mediator, or whether the age signal is confounded by known biomarkers, we tested competing predictors of ICI benefit before drawing mechanistic conclusions (Fig. 5). Tumor mutational burden in TCGA-OV showed no association with age group (Wilcoxon p = 0.663, n = 195), excluding neoantigen load as an operative variable. Homologous recombination status in our clinical cohort failed to predict response (Fisher exact p = 1.0, n = 19), though this analysis is underpowered at n = 20; the HRD–STING linkage via PARP inhibitor sensitivity^5-6^ makes a modest effect biologically plausible and motivates the R01-scale validation cohort. Germline BRCA status was insufficient alone, as 38% of BRCA wildtype patients responded to ICI. cGAS expression in TCGA-OV was unchanged with age (Wilcoxon p = 0.771), localizing the defect downstream of DNA sensing. BRCA1 promoter methylation in the TCGA-OV treatment-naive subset (n = 273 patients from 282 arrays, all confirmed primary tumor via GDC sample-type verification) did not correlate with age (Spearman ρ=−0.030, p = 0.62). An eight-gene immune checkpoint expression panel (PDCD1, CD274, CTLA4, HAVCR2, LAG3, TIGIT, VSIR, PDCD1LG2) across the three transcriptomic cohorts (24 Wilcoxon tests) yielded no gene passing multiple-testing correction (0/24 pass BH FDR < 0.05). VSIR showed a nominal TCGA-OV signal (raw p = 0.0025, BH FDR = 0.059, higher in younger patients) that did not survive correction and did not replicate in the two microarray cohorts; the age-dependent immune configuration is therefore not attributable to differential VSIR expression.

Only STING1 satisfied the pre-specified criteria for a primary mediator: reproducible reduction across three independent platforms in the a priori direction, mechanistic consistency with an attenuated rather than absent sensing pathway (given intact upstream cGAS), concordance with the parallel reduction of IFI16, and biological plausibility from established pan-cancer tumor-intrinsic STING silencing. These converging criteria support STING1 attenuation, with IFI16 as secondary contributor, as the discrete molecular lesion accounting for the age-dependent BRCA–immune reversal and the clinical ICI response phenotype.

## Discussion

This study identifies patient age as an active biological determinant of the ovarian cancer immune microenvironment. Across 761 patients pooled from four independent cohorts on three orthogonal transcriptomic platforms, we show that the direction of the BRCA–immune relationship is reversed by patient age, with zero statistical heterogeneity between cohorts. This reversal is mediated by a discrete molecular lesion — selective attenuation of the innate immune signal transducers STING1 and IFI16, with the upstream sensor cGAS preserved — and propagates to downstream immunometabolic effectors including IDO1. In a fully clinically annotated ICI-treated cohort (n = 20), this molecular configuration is accompanied by a five-fold difference in objective ICI response rate between patients ≥ 60 years (75%) and patients < 50 years (14%), independent of BRCA status and HR status.

The mechanism identified here is discrete and localizable rather than generic. cGAS sensing is preserved while STING1 and IFI16 transduction is attenuated. This dissociation places the age-associated lesion downstream of DNA damage recognition and upstream of interferon output, and distinguishes the phenomenon from the diffuse decline of immune function conventionally associated with immunosenescence^15^. Together with the pan-cancer literature on recurrent tumor-cell–intrinsic STING silencing^13^, this pattern is consistent with tumor-compartment–specific epigenetic silencing as a candidate mechanism, though this must be confirmed at compartment resolution.

The paradigm that emerges is qualitatively distinct from the prevailing framework in ovarian cancer immunotherapy. The dominant model — rooted in the empirical observation of low ICI response rates — has been that ovarian cancer is intrinsically refractory to immune checkpoint blockade^2-4^. Our data are incompatible with this framing. The median age of trial participants in prior ovarian cancer ICI studies (approximately 60 years) places typical enrollment squarely within the peri- and early post-menopausal transition range that we deliberately excluded from primary analysis, the same range in which pre- and post-menopausal immune states mix in unknown proportions. When we instead compare biologically distinct pre-menopausal and post-menopausal patients, the age effect is not subtle: a five-fold response rate difference emerges, together with molecular signatures replicated across three independent transcriptomic cohorts. Prior trials therefore appear to have tested ICI predominantly in the wrong subpopulation, while the older post-menopausal group in which the response is concentrated has been underrepresented at enrollment. The prevailing view that ICI is ineffective in ovarian cancer is, on the data presented here, a trial-design artifact rather than a biological property of the disease. Consistent with this reading, KEYNOTE-100 (n = 376), the largest single-agent ICI trial in this disease, reported a null age subgroup analysis in its primary results^2^ — a result expected given that any dichotomous cutoff near the median enrollment age (~ 60 years) mixes pre- and post-menopausal patients across the transition zone. Prior efforts to link aging biology to ICI response have been correlative and pan-cancer^16^, or have used computational aging scores that do not implicate the cGAS–STING axis^17^; studies of the aging ovarian tumor microenvironment have identified FOXP3^+^ regulatory T cell expansion as an effector^18^ but attribute the phenotype to adaptive immunosuppression rather than to innate signal transduction. Immunosenescence reviews have predicted a diffuse decline in ICI benefit with age driven by T cell exhaustion^15^ — the opposite of what we observe in this disease. Our findings suggest an alternative model in ovarian cancer: rather than uniformly impaired immunity, older patients have a molecularly distinct tumor immune microenvironment defined by STING1/IFI16 attenuation that remains responsive to checkpoint blockade.

The implication for clinical trial design is immediately actionable. Patient age is recorded on every individual in every clinical trial and requires no additional assay; age-stratified enrollment and pre-specified age subgroup analyses could be implemented in the next generation of ovarian cancer ICI trials without any change to trial operations, and prior trials should be re-analyzed for age-stratified response. Beyond enrollment, the observed age-dependent STING1/IFI16 attenuation provides a rationale for evaluating STING agonist strategies specifically in older ovarian cancer patients, in whom this transduction lesion is the operative signal.

Several limitations should be noted. The ICI-treated clinical cohort is single-institution and small (n = 20); while the 75% versus 14% response rate is statistically significant (Fisher p = 0.041), the finding requires prospective validation in a larger, multi-institutional cohort, and expansion through a tissue microarray of the USC ovarian cancer cohort is planned. The transcriptomic cohorts are cross-sectional and do not directly demonstrate that age-associated decline in STING1 within an individual patient drives immune remodeling. Finally, bulk transcriptomes cannot resolve whether STING1 and IFI16 attenuation occurs in the tumor epithelial compartment or in infiltrating cells; because tumor-cell–intrinsic STING silencing is compartment-specific in pan-cancer datasets^13^ and stromal cGAS–STING is hyperactive during inflammaging, compartment-resolved analysis is a critical next step.

The operative question in ovarian cancer immunotherapy may not be whether ICI works but in whom — a question patient age can begin to answer at enrollment today, and that spatial immune architecture and mechanistically informed molecular readouts may ultimately answer through a deployable clinical assay.

## Methods

### Clinical cohort

The USC/LAGMC cohort comprised 20 women with histologically confirmed ovarian cancer treated with immune checkpoint inhibitor therapy at USC Norris Comprehensive Cancer Center and Los Angeles General Medical Center between 2018–2023. All specimens were formalin-fixed paraffin-embedded (FFPE). Clinical metadata included age at diagnosis, TNM stage, germline or somatic BRCA1/BRCA2 mutation status, HR status (homologous recombination proficient, HR-deficient by germline/somatic BRCA mutation, or HR-deficient by mechanism other than germline BRCA), ICI regimen, and progression-free interval (PFI, time from ICI initiation to documented progression). Response was classified using a pre-specified durable-benefit definition based on the PFI: PFI ≥ 365 days = Responder, PFI < 365 days = Non-responder. This 365-day threshold captures durable clinical benefit rather than transient anatomic tumor shrinkage. BRCA classification distribution: wildtype n = 8, germline or somatic mutant n = 9, HR-deficient without germline BRCA mutation n = 3. The pre-specified age stratification was Old (≥ 60 years, n = 8) versus Young (< 50 years, n = 7), with 50–59-year patients (n = 5) excluded from primary comparisons^12^. Use of these specimens was approved by the USC Institutional Review Board as exempt under 45 CFR 46.104(d)(4) (protocol HS-23-00282).

### Public transcriptomic cohorts

Four independent public ovarian cancer transcriptomic cohorts were used. TCGA-OV^8^ (RNA-seq; NCI/GDC) provided n = 226 tumors with age at diagnosis after filtering for high-grade serous histology and available BRCA pathway gene expression, and n = 613 tumors on the Illumina HumanMethylation27 (HM27) BeadChip subset used for BRCA1 promoter methylation analysis. GSE9891 (Peter MacCallum, Melbourne)^9^ provided n ≈ 285 tumors profiled on Affymetrix HG-U133 Plus 2.0. GSE140082 (AGO-OVAR cohort)^10^ provided n = 380 tumors profiled on the Illumina HumanHT-12 WG-DASL V4.0 R2 BeadChip. The Medical University of Innsbruck (MUI) cohort^11^ provided n = 60 tumors profiled by bulk RNA-seq. Processed expression data and clinical annotations were obtained from the respective consortium repositories (Firebrowse for TCGA-OV; Gene Expression Omnibus for GSE9891 and GSE140082; Zenodo DOI 10.5281/zenodo.10251467 for the MUI cohort).

### Age stratification and covariate handling

For all analyses, patients were dichotomized into Young (< 50 years) and Old (≥ 60 years) groups, with 50–59 year patients excluded from primary comparisons by pre-specified design. Menopausal status was not directly ascertained in the public transcriptomic cohorts (only chronological age was available); we therefore used age as an approximate proxy for reproductive/menopausal status, excluding the intervening range because it encompasses the peri- and early post-menopausal transition during which reproductive and immune status vary substantially between women of the same chronological age^12^. The exclusion is methodological: 50–59 year patients were processed identically and contribute to descriptive characterization; only the primary inferential tests restrict to the boundary age groups.

### BRCA pathway activity and immune infiltration scoring

BRCA pathway activity was scored per patient by Gene Set Variation Analysis (GSVA) using the Reactome BRCA/HRR gene set. Immune infiltration was scored using CIBERSORTx and LM22 signatures for cell-type deconvolution, and by an interferon-stimulated gene signature score. Tumor purity was estimated by ESTIMATE for adjustment. HR status in the USC/LAGMC clinical cohort was assigned by the treating team as a categorical classification (HR-proficient, HR-deficient by germline or somatic BRCA1/BRCA2 mutation, or HR-deficient by mechanism other than germline BRCA) based on clinical genomic testing performed per institutional standards; a continuous HRD score was not available for this cohort. For MUI Innsbruck, where a BRCAness classifier probability was the available annotation rather than a GSVA pathway score, we used -BRCAness_prob as the BRCA-pathway predictor so that higher values consistently indexed higher HRR-pathway activity across all cohorts.

### Age-by-BRCA interaction meta-analysis

For each transcriptomic cohort, a linear model of immune infiltration score against BRCA pathway GSVA score, patient age group (Old vs. Young), and their interaction was fit, adjusting for tumor purity, stage where available, and cohort-specific covariates. The age-by-BRCA interaction coefficient (b) and its standard error were extracted from each cohort. Meta-analysis was performed under a random-effects model (DerSimonian–Laird) using the metafor R package. Between-cohort heterogeneity was quantified by I^2^. Leave-one-out sensitivity analysis was performed to assess robustness.

### Age-dependent gene expression analysis

Age-dependent expression of individual pathway genes (cGAS/MB21D1, STING1/TMEM173, IFI16, IDO1) was tested by Wilcoxon rank-sum test comparing Old versus Young groups within each cohort. False discovery rate correction was applied within each cohort using the Benjamini–Hochberg method. Spearman rank correlation was used for continuous age-expression relationships. Cross-cohort concordance was assessed by direction of effect and effect-size magnitude.

### Competing hypotheses

Five pre-specified competing predictors were tested: tumor mutational burden (in TCGA-OV, tested against age group), HR status (in the USC/LAGMC clinical cohort, tested against response; TMB not available), germline BRCA status alone (in USC/LAGMC), cGAS expression (in TCGA-OV, tested against age), BRCA1 promoter methylation (TCGA-OV HM27 subset, treatment-naive n = 273 patients from 282 arrays, methylation β-values tested against age), and an eight-gene immune checkpoint expression panel (PDCD1, CD274, CTLA4, HAVCR2, LAG3, TIGIT, VSIR, PDCD1LG2) tested across three transcriptomic cohorts (TCGA-OV, GSE9891, GSE140082) with Benjamini–Hochberg FDR correction across the 24 tests.

### Statistical software

Primary statistical analyses (meta-analysis, per-cohort linear models, pathway scoring, differential expression, correlation, and multiple-testing correction) were performed in R version 4.3. Meta-analysis: metafor v4.6 (REML random-effects model). Pathway scoring: GSVA v1.50. Immune deconvolution: CIBERSORTx (web implementation). FDR correction: Benjamini–Hochberg method as implemented in stats::p.adjust. Visualization of tabular and cohort-level outputs: ggplot2 and ComplexHeatmap. Publication figure assembly used Python 3.11 with matplotlib 3.10, pandas 2.2, numpy 1.26, scipy 1.14, and statsmodels 0.14. Reproducible analysis code and processed per-patient data are made available as described in Code availability, below.

## Figures and Tables

**Table 1.** Ovarian cancer transcriptomic cohorts used in this study.

TCGA-OV — RNA-seq (Illumina HiSeq); NCI/Genomic Data Commons; n=226 with age and BRCA pathway data; also n=613 on Illumina HumanMethylation27 BeadChip subset for methylation analysis.
GSE9891 — Affymetrix HG-U133 Plus 2.0; Peter MacCallum Cancer Centre, Melbourne (Australian Ovarian Cancer Study); n≈285.
GSE140082 — Illumina HumanHT-12 WG-DASL V4.0 R2 BeadChip (FFPE); AGO-OVAR (Kommoss et al.); n=380.
MUI Innsbruck — Bulk RNA-seq (Gronauer et al.); Medical University of Innsbruck; n=60.

## Data Availability

All public transcriptomic data used in this study are available through their respective repositories. TCGA-OV expression, methylation, and clinical data are available through the NCI Genomic Data Commons (dbGaP accession phs000178) with processed expression data mirrored at Firebrowse. GSE9891 (Australian Ovarian Cancer Study, Peter MacCallum) is available at Gene Expression Omnibus (accession GSE9891). GSE140082 (AGO-OVAR) is available at Gene Expression Omnibus (accession GSE140082). The Medical University of Innsbruck (MUI) cohort is available at Zenodo (DOI 10.5281/zenodo.10251467). De-identified processed per-patient data files derived from the public cohorts and sufficient to reproduce every figure derived from public data are provided in the code repository described in Code availability, below — including within-cohort z-scored BRCA/HRR pathway and T-inflamed GEP scores (Fig. 2c), per-gene expression values for STING1, IFI16, and cGAS across the three transcriptomic cohorts (Fig. 3), and per-patient competing-predictor values from TCGA-OV (Fig. 5b-c). Clinical metadata for the USC/LAGMC ICI-treated cohort (n = 20) cannot be shared publicly due to institutional data-sharing restrictions and the risk of re-identification in a small single-institution cohort. Aggregate summary statistics for this cohort are reported in full in Fig. 1, Fig. 5a, and the Results text. Requests for access to the individual-level USC/LAGMC clinical data may be directed to the corresponding author and are subject to institutional review and a data-sharing agreement with USC.

## Code availability

All analysis code required to reproduce the public-cohort results reported in this study will be publicly available at github.com and archived at Zenodo with a citable DOI at the time of publication. The repository is organized into three components. (i) R analysis pipeline: scripts implementing the age-by-BRCA interaction meta-analysis (metafor v4.6, REML random-effects model), per-cohort linear models, GSVA pathway scoring (GSVA v1.50 with the Reactome BRCA/HRR gene set), CIBERSORTx immune deconvolution wrappers, per-gene Wilcoxon rank-sum tests with Benjamini–Hochberg FDR correction, Spearman correlation analyses for continuous age–expression relationships, and TCGA-OV BRCA1 methylation analysis in the treatment-naive subset with GDC sample-type verification. Principal script: meta_analysis_BRCA_Age_Immune.R. (ii) Python figure generation: scripts producing all main-text figures from processed per-patient data (make_fig2.py — meta-analysis forest plot, per-cohort scatterplots, leave-one-out sensitivity; make_fig3.py — STING1/IFI16/cGAS boxplots and pathway schematic; make_fig5.py — competing-hypothesis panels and summary table). Dependencies: matplotlib 3.10, pandas 2.2, numpy 1.26, scipy 1.14, statsmodels 0.14. (iii) Processed per-patient data files derived from public cohorts and sufficient to regenerate every figure derived from public data: scatter_data_fig2c.csv, gene_expression_fig3.csv, tcga_ov_competing_predictors.csv, checkpoint_panel_expression.csv, meta_analysis_results.rds (metafor output object), and tcga_ov_methylation_verification.log (GDC sample-type verification record). Individual-level clinical data from the USC/LAGMC cohort is not included in the repository per institutional data-sharing restrictions (see Data availability). A top-level README documents the full reproduction workflow from raw public data through final figures. All code is released under the MIT License. The repository additionally includes session-info records (R sessionInfo() and Python pip freeze output) documenting exact package versions used in the analyses reported here.
